# Comparative Evaluation of the Nutritional, Antinutritional, Functional, and Bioactivity Attributes of Rice Bran Stabilized by Different Heat Treatments

**DOI:** 10.3390/foods10010057

**Published:** 2020-12-28

**Authors:** Maria Irakli, Athina Lazaridou, Costas G. Biliaderis

**Affiliations:** 1 Institute of Plant Breeding & Genetic Resources, Hellenic Agricultural Organization—Demeter, Thermi, 57001 Thessaloniki, Greece; 2 Department of Food Science and Technology, School of Agriculture, Aristotle University of Thessaloniki, P.O. Box 235, 54124 Thessaloniki, Greece; athlazar@agro.auth.gr (A.L.); biliader@agro.auth.gr (C.G.B.)

**Keywords:** rice bran, stabilization, antioxidants, functional properties, bioactives, anti-nutritional components

## Abstract

The objective of this study was to evaluate the effects of different stabilization treatments—namely, dry-heating, infrared-radiation, and microwave-heating—on the nutritional, antinutritional, functional, and bioactivity attributes of rice bran (RB). Among the heating treatments, infrared-radiation exerted the strongest inactivation, resulting in 34.7% residual lipase activity. All the stabilization methods were found to be effective in the reduction of antinutrients, including phytates, oxalate, saponins, and trypsin inhibitors. No adverse effect of stabilization was noted on chemical composition and fatty acid profile of RB. Instead, stabilization by all heat treatments caused a significant decrease of vitamin E and total phenolics content in RB; the same trend was observed for the antioxidant activity as evaluated by the DPPH test. The antioxidant activity, as evaluated by ABTS and FRAP tests, and water absorption capacity were improved by the stabilization of RB, whereas the oil absorption capacity and emulsifying properties decreased. Microwave-heating enhanced the foaming properties, whereas infrared-radiation improved the water solubility index and swelling power of RB. Consequently, treatment of RB with infrared-radiation has a potential for industrialization to inactivate the lipase and improve some functional properties of this material for uses as a nutraceutical ingredient in food and cosmetic products.

## 1. Introduction

Rice bran (RB) is an agro-industrial by-product of rice kernel dry milling and consists mainly of the germ, pericarp, aleurone, and sub-aleurone layers [1]. It is a natural source of protein (14–16%), fat (12–23%), crude fiber (8–10%), other carbohydrates, vitamins, minerals, essential unsaturated fatty acids, and antioxidants, such as phenolics, tocopherols, tocotrienols, and γ-oryzanol with well-known beneficial effects in human health [2]. The predominant bioactive compound in the RB is γ-oryzanol due to its antioxidant [3], hypocholesterolemic [4], anti-inflammatory [5], anti-cancer [6], and anti-diabetic properties [7]. Furthermore, RB is gaining increased interest in the food, nutraceutical, and pharmaceutical industries, due to its high nutritional value, low cost, easy availability, high bioactivity potential, and the associated health benefits [2].

Despite the presence of various bioactives, the majority of RB is under-utilized as animal feed, fertilizer, or fuel in many developing countries. In Asian countries, it is used successfully for the production of RB oil with many health benefits. The main limitation in food industry applications is the requirement for quick stabilization of the RB, in order to reduce the antinutrients present and deactivate the action of lipase present in the outer layers of rice grain, which is primarily responsible for the hydrolysis of triglycerides into glycerol and free fatty acids following the milling process of the rice grains [8]. Immediately after milling, the free fatty acids level is increased, and the RB is unsuitable for human consumption, owing to rancid flavor and soapy taste. Moreover, lipoxygenase and peroxidase also contribute to the rancidity of RB although to a lesser extent [9].

Several stabilization treatments including dry and moist heating [10], extrusion cooking [11], microwave-heating [12,13], infrared-radiation [14,15], ohmic heating [16], parboiling or hydrothermal treatment [17], other physical methods of stabilization or refrigeration [18], enzymatic treatment [19], and chemical methods [20] have been employed immediately after milling to prevent the development of rancidity in RB. The selection of an optimized stabilization method is a crucial point for the food industry in order to ensure high component yield, low cost, and limited loss of bioactive constituents. 

As a process, dry-heating is simple, convenient, and amenable to industrialization [21]. On the other hand, microwave-heating and infrared radiation are considered as alternative energy sources with high heat efficiency over shorter processing times without affecting RB quality. Microwave stabilization has been referred to as a quick heating method with high efficiency to inactivate lipase along with a better retention of bioactive compounds [12,22]. Similarly, infrared-radiation constitutes an alternative processing method to achieve efficient drying along with lipase inactivation in RB without affecting its quality [14,15,23]. In recent years, infrared-radiation heating has become an important technique in the food processing industry, because of its numerous advantages, such as the low capital cost and low energy cost, the simplicity of the required equipment, a lower drying time, the high quality of dried products, easy control of the process parameters, a uniform temperature distribution, and the clean operational environment, as well as space savings and easy accommodation with convective, conductive, and microwave heating [24]. As for the microwave heating, there are several major drawbacks that limit its application as the sole heating method for the drying process due to the high start-up costs, relatively complicated technology as compared to conventional convection drying despite the shorter drying time, improved product quality, and flexibility [25].

Although the efficiency of the microwave and infrared-radiation heating methods in stabilizing the RB, in relation to nutritional and bioactives profile, has been extensively studied by many researchers, to date, there are no reports dealing systematically with the effects of stabilization methods on functional properties from the technological point of view (physicochemical properties of RB) and their impact on anti-nutritional components. Relevant research works in this respect refer to protein isolates extracted from RB stabilized with different methods [26,27].

Therefore, the present study aimed to investigate the effects of dry-heating, microwave and infrared-radiation heating on the nutritional, antinutritional, and bioactive components of stabilized RB as well as on its functional properties and antioxidant capacities.

## 2. Materials and Methods

### 2.1. Sample Preparation

Fresh RB (10.93% moisture content) was collected from a local rice mill (Megas Alexandros, Sindos, Thssaloniki, Greece), and it was stabilized immediately using three heating processes as described below: (i) infrared-radiation: RB was transferred in a custom-made device consisting of a shaking aluminum tray, a radiator with two infrared lambs and a connected thermostat, as described by Irakli et al. [15] and it was heated at 140 °C for 15 min, (ii) dry-heating: RB was spread in thin layers to open pans and then was heated in an oven at 150 °C for 40 min and (iii) microwave-heating: RB was moistened up to 21% and then was placed in a glass plate (spread out in thin layer) and subjected to heating for 2 min at 650 W, corresponding to an approximately temperature of 160 °C. The stabilized RB samples were subsequently cooled at ambient temperature, packed in plastic containers, and stored at 4 °C for further use. An unstabilized RB sample was used as a control for comparative purposes, and it was stored under the same conditions as the stabilized RB samples.

### 2.2. Color Measurements

Color was measured using a colorimeter (HunterLab, model MiniScan XE Plus, Reston, VA, USA), following the CIE system defined by the L*, a*, and b* coordinates. The total color differences (ΔE) between the control and stabilized RB samples were calculated using the equation: ΔE = (ΔL*^2^ + Δa*^2^ + Δb*^2^)^1/2^ [28]. 

### 2.3. Proximate Analysis

Moisture, protein, ash, and fat content were determined according to official methods [29]. Protein content was estimated by the Kjeldahl method, ash was determined by the dry ashing procedure, and fat content was determined using a Soxhlet apparatus, whereas total carbohydrates were calculated by difference. 

### 2.4. Fatty Acid Composition

The fatty acid composition of the RB samples extracted with ether was determined using gas chromatography with flame ionization detection (Model Varian CP-3800, Middelburg, The Netherlands) based on the AOAC 996.06 method [30]. Fatty acid methyl esters were identified by comparison of their retention times with those of external standards (Supelco 37 Component FAME Mix) and the amount of individual fatty acids was expressed as percentages (%) of the total fatty acids determined. 

### 2.5. Free Fatty Acids Content

Free fatty acids contents of the lipid fraction of RB samples were determined using a standard titration method [31], and the results were calculated as oleic acid equivalent, which was expressed as a percentage of total lipids.

### 2.6. Lipase Assay

The lipase activity in RB samples was determined according to Rose and Pike [32] with some modification. Briefly, 1 g of defatted RB was weighed into each of two test tubes: one blank and one sample. Then, 400 μL of olive oil and 200 μL of distilled water were added to both tubes and mixed. The lipids from the blank were immediately extracted with 5 mL of hexane (2 times), the supernatants after centrifugation at 1000× *g* for 5 min were pooled and evaporated using a rotary vacuum evaporator, and the residue was redissolved in 4 mL of isooctane. The other test tube (sample) was incubated for 4 h at 40 °C. After incubation, lipids were extracted as described for the blank, and both extracts were used for the lipase assay. An aliquot of 0.75 mL isooctane extract was mixed with 0.5 mL of 3% (*v*/*v*) pyridine in 5% (*w*/*v*) aqueous cupric acetate, the mixture was shaken for 1 min, centrifuged for 1 min, and the absorbance of supernatant was read at 715 nm and quantified against an external standard curve of oleic acid. The lipase activity was expressed as units per g RB (U/g), where 1 U was defined as the micromoles of fatty acid liberated per h, according to Equation (1):

*Lipase activity* = 1000 (4 + *ν*) (*As* − *AB*)/*εtls* (1)

where *v* = volume of olive oil added (mL), *As* = absorbance of sample after incubation at 715 nm, *AB* = absorbance of blank at 715 nm, *ε* = molar absorptivity of oleic acid at 715 nm (M^−1^·cm^−1^), *t* = incubation time (h), *l* = path length (1 cm for a standard cuvette), and *s* = sample weight (g).

### 2.7. Bioactive Components

*Free phenolic compounds* were extracted twice from 0.25 g sample with 5 mL of 60% aqueous ethanol in an ultrasound bath at room temperature for 10 min, followed by centrifugation at 10,000 rpm for 10 min at 4 °C. *Bound phenolic compounds* were recovered after the alkaline hydrolysis of the remained residue with 4N NaOH for 90 min under sonication, followed by acidification to pH 2.0 with concentrated HCl and finally extraction with ethyl acetate. The ethyl acetate fraction was vacuum-evaporated at 40 °C, and the residue was reconstituted in 4 mL of 70% aqueous methanol. Both free and bound fractions were stored at −25 °C until analysis.

*Total phenolic content* (TPC) of extracts was performed using the Folin–Ciocalteu’s method according to Singleton et al. [33]. Briefly, extracts of 0.2 mL were mixed with 0.8 mL Folin–Ciocalteu reagent, 2 mL of sodium carbonate (7.5% *w*/*v*) solution, and distilled water until reaching a final volume of 10 mL. The absorbance at 725 nm was recorded after incubation for 60 min. The results were expressed as mg of gallic acid equivalents per g of sample on a dry basis (mg GAE/g dw).

*Tocopherols and tocotrienols contents* were determined as follows: 0.2g of sample was sonicated twice with 2.5 mL ethanol for 10 min, and the extract was collected after centrifugation at 1500× *g* for 10min. The combined supernatants were evaporated under the flow of nitrogen, the remaining residue was redissolved in 1 mL of a mixture acetonitrile/methanol (85:15, *v*/*v*), and finally, a 20 μL aliquot was injected into an HPLC system (Agilent Technologies, 1200 series, Urdorf, Switzerland) equipped with a YMC C_30_ column (250 × 4.6 mm id, 3 μm, MZ Analysentechnik, Mainz, Germany); the chromatographic conditions were as described by Irakli et al. [34].

*γ-oryzanol content* was determined in the same extract as with tocol analysis. The separation was carried out using a Target C_18_ (4.6 × 150 mm, 5 μm, MZ Analysentechnik, Mainz, Germany) column at 30 °C, with mobile phase consisted of acetonitrile/methanol/dichloromethane (40:45:15, *v*/*v*/*v*) under isocratic conditions at a flow rate of 1.5 mL/min. γ-oryzanol was detected at 330 nm. Quantitation was based on a linear calibration curve of the sum of the areas of four curves of γ-oryzanol standard, namely cycloartenyl ferulate, 24-methylene cycloartanyl ferulate, campesteryl ferulate and sitosteryl ferulate, as were analyzed by HPLC and confirmed by nuclear magnetic resonance (NMR) and MS by others researchers [35].

### 2.8. Antinutritional Composition

*Oxalate content* was determined using the titration method described by Oyeyinka et al. [36]. Firstly, 1 g of sample was extracted with 75 mL of 3M sulfuric acid under continuous mechanical stirring for 1 h, followed by filtration. Then, 25 mL of the filtrate was heated at 70 °C and was titrated steadily against 0.05 M potassium permanganate, until an extremely faint, pale pink end point color persistence was observed for 10 s. Oxalate content was calculated by the equation: *Oxalate* (*mg*/*g*) = *1.33 x titer value*. The results were expressed on a dry basis.

*Phytate content* was evaluated using the method as described by Wheeler and Ferrel [37]. Firstly, 1 g of sample was macerated with 50 mL of 3% trichloroacetic acid (TCA) for 30 min with mechanical shaking. After centrifugation, a 10 mL aliquot of supernatant was mixed with 4 mL of FeCl_3_ (2 mg ferric ion per mL in TCA), and the mixture was boiled for 45 min. After centrifugation, the precipitate was washed with 20–25 mL 3% TCA and was re-boiled for another 10 min. The washing was repeated with water, and the precipitate was dispersed in few mL water and 3 mL of 1.5N NaOH, and water was then added until the final volume of 30 mL. The suspension was boiled for 30 min, followed by filtration and washing with hot water, and the precipitate was dissolved with 40 mL of hot 3.2N HNO_3_ into a 100-mL volumetric flask that was filled with the washing water; then, a 5 mL aliquot of the above extract was transferred in a 100-mL volumetric flask containing 70 mL water and 20 mL 1.5N KSCN was added, filled with water, and the absorbance was measured at 480 nm within 1 min. Iron content from Fe(NO_3_)_3_ was measured via a standard curve, and phytate phosphorus was calculated from the iron results assuming a 4:6 iron/phosphorus molecular ratio with the results being expressed on dry basis.

*Total saponin content* was determined using a spectrophotometric method, as described by Hierro et al. [38]. Firstly, 0.5 g of sample was extracted with 10 mL of absolute ethanol in an ultrasonic bath for 20 min at 70 °C. Then, the mixture was centrifuged at 4000 rpm for 10 min, and the above procedure was repeated. Combined supernatants were dried under vacuum, and the residue was reconstituted in 5 mL absolute ethanol. Aliquots of 250 μL were mixed with 250 μL of freshly prepared vanillin in ethanol (0.8%, *w*/*v*) and 2.5 mL of sulfuric acid in water (72%, *v*/*v*). Mixtures were vortexed, heated at 60 °C for 10 min, cooled in ice for 5 min, and the absorbance was recorded at 560 nm against the control sample containing ethanol. Results were expressed as mg diosgenin equivalent per g RB sample on dry basis.

*Trypsin inhibitor activity* was estimated by the method of Kakade et al. [39] as follows: 1 g of sample was extracted with 50 mL of 0.01 NaOH with continuous shaking for 1 h. The pH of the suspension was 9.5 to 9.8, and the suspension was diluted to the point that 1 mL produces trypsin inhibition of 40 to 60%. Then, portions of 0, 0.6, 1.0, 1.4, and 1.8 mL of the suspension were transferred into a double set of test tubes and adjusted to 2 mL with water. After the addition of 2 mL of trypsin (0.2 mg/mL), the tubes were boiled at 37 °C and 5 mL of BAPA solution (40 mg benzoyl·d l·arginine-p-nitroanilide hydrochloride dissolved in 1 mL of dimethyl sulfoxide and diluted to 100 mL with Tris-buffer, 0.05M, pH 8.2, containing 0.02M CaCl_2_) was added, and the mixture was incubated for exactly 10 min. The reaction was terminated with 1 mL 30% acetic acid, and the absorbance was recorded at 410 nm against the reagent blank that was prepared by adding 1 mL of 30% acetic acid before the addition of BAPA. Trypsin inhibition activity is expressed in terms of trypsin units inhibited (TUI) taking into account that 1 TU is arbitrary defined as an increase of 0.01 absorbance units at 410 nm per 10 mL of extract on dry basis.

*Tannins content* was estimated as follows: Aliquots of about 400 μL of the phenolic extract prepared as described above were treated with polyvinylpolypyrrolidone (40 mg, 100 mg/mL) at 4 °C for 20 min. After centrifugation at 10,000 rpm (4 °C, 10 min), non-tannin phenolics (supernatant) were determined as above by the Folin–Ciocalteu method [40]. Total tannins were calculated by subtracting non-tannin phenolics from total phenolics.

### 2.9. Antioxidant Activity Assays

Three assays were employed to determine the antioxidant activities of the RB extracts: 2,2’-azinobis-(3-ethylbenzothiazoline-6-sulphonate) radical (ABTS·+) scavenging activity (ABTS assay) [41], 1,1-diphenyl-2-picrylhydrazyl radical scavenging activity (DPPH assay) [42] and ferric-reducing antioxidant power (FRAP) [43]. Trolox was used as the standard compound for calibration curves and the results were expressed in mg of trolox equivalents (TE) per g of RB on dry basis (mg TE/g dw).

### 2.10. Bulk Density

Bulk density (BD) of RB samples was determined by the method described by Kaushala et al. [44]: 25 g of flour was gently filled into 50 mL graduated cylinders that were previously tared. The bottom of each cylinder was gently tapped several times, and the volume was measured. BD was calculated as the weight of the sample per unit volume of sample (g/mL).

### 2.11. Functional Properties Determination

The water absorption capacity (WAC) of the RB samples was determined according to Abebe et al. [45]: RB samples were dispersed in distilled water and diluted to 10 g/mL. The dispersions were held at room temperature for 30 min with continuous stirring and followed by centrifugation for 30 min at 4000× *g*. Then, the supernatant was weighed, and the results were expressed as g of water retained per g of RB.

Oil absorption capacity (OAC) was determined according to Kaushala et al. [44]: A RB sample (0.5 g) was mixed with 6 mL of corn oil in pre-weighed tubes and centrifuged for 25 min at 3000× *g*. After stirring for 1 min with a thin brass wire, the tubes were allowed for 30 min and then were centrifuged for 25 min at 3000× *g*. The separated oil was removed with a pipette, and the tubes were inverted for 25 min to drain the oil prior to reweighing. The OAC was expressed as g of oil bound per g of the RB.

The water absorption index (WAI) and water solubility index (WSI) were measured according to Abebe et al. [45]. First, 2.5 g of RB sample was mixed with 30 mL of distilled water, vortexed, and cooked for 10 min in a 90 °C water bath. After centrifugation at 4000× *g* for 10 min, the supernatant was weighted and evaporated overnight at 110 °C to determine the soluble solids, and the sediment was weighed. The WAI was calculated by weighing the sediment and expressed as g of absorbed water per g of sample. WSI was calculated from the amount of soluble solids recovered from the supernatant divided by the sample weight × 100. The swelling power (SP) was calculated by dividing the weight of sediment per the difference between the initial sample weight and the amount of dry solids of the supernatant (g/g).

The foaming capacity (FC) and foaming stability (FS) were determined using the method described by Kaushala et al. [44]. The dispersions of the sample (1.5 g in 50 mL of distilled water) were homogenized, using an ultrasonic probe (Sonoplus, model HD 4100, Berlin, Germany) at high speed for 2–3 min. The suspension was immediately transferred into a graduated cylinder, and the homogenizer cup was rinsed with 10 mL of distilled water, which was then added to the graduated cylinder. The volume was recorded before and after whipping. FC was calculated by dividing the difference between the volume after and before the whipping per the volume before whipping × 100. Foam volume changes in the graduated cylinder were recorded at an interval of 60 min of storage in order to estimate the FS.

Emulsifying activity (EA) was determined by the method of Nazck et al. [46]. First, 3.5 g sample of RB was homogenized for 30 s with 50 mL of water using an ultrasonic probe (Sonoplus model HD 4100, Berlin, Germany) at high speed. After the addition of 25 mL of corn oil, the mixture was again homogenized for 90 s. The emulsion was centrifuged at 1100× *g* for 5 min. The EA was calculated by dividing the volume of the emulsified layer by the volume of emulsion before centrifugation × 100. The emulsion stability (ES) was determined by heating the above emulsion at 85 °C for 15 min, after which it was cooled and centrifuged. The ES was expressed as a percentage of the EA remaining after heating.

All results were expressed on dry basis to avoid the effect of different water content in the samples.

### 2.12. Statistical Analysis

Values were reported as the mean ± standard deviation of triplicate measurements. All parameters were subjected to one-way analysis of variance (ANOVA), and when ANOVA revealed significant differences between means, a Tukey’s test at *p* < 0.05 was used to separate means by using the Minitab 17 (Minitab Inc., State College, PA, USA) software.

## 3. Results and Discussion

### 3.1. Free Fatty Acids and Lipase Activity

**Figure 1:**
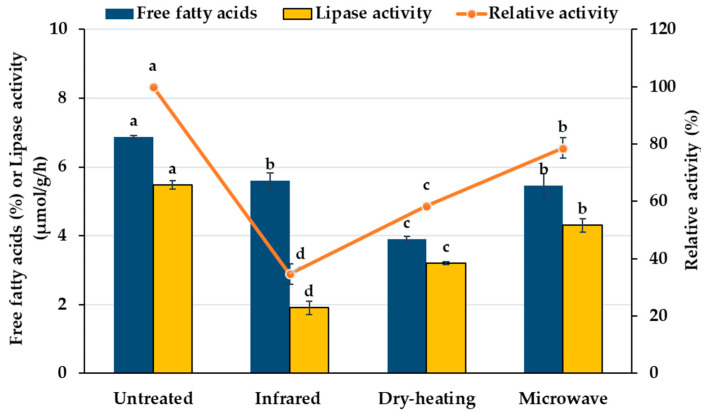
Effect of three different heating treatments (infrared-radiation, dry-heating, and microwave-heating) on free fatty acids content and relative lipase activity of RB samples; values followed by the same letter for the same estimated parameter are not significantly different (*p* > 0.05).

Heating treatments are the most widely used methods of RB stabilization. Stabilized RB offers multiple benefits including better quality characteristics and extended product shelf-life on storage, thus providing a wider range of RB fortified products for consumers. Novel technologies in food preservation have gained increased industrial interest as they are more environmentally and economically sustainable compared to conventional preservation methods. In this context, the effects of three different heating treatments, such as dry-heating, infrared-radiation, and microwave-heating, on the stabilization of RB were examined in the present study on a comparative basis. The applied conditions (temperature and time duration) for each heating treatment were selected according to preliminary results based on response surface methodology using relative lipase activity as a probe for the effectiveness of the treatment (data not shown). The free fatty acids (FFA) content and lipase activity of untreated and heat-treated RB samples were determined after 1 day of storage at room temperature. As shown in Figure 1, significant decreases (*p* < 0.05) on FFA content were observed in the heat-treated RB samples by all three methods of heat treatment employed. Among the heating treatments, dry-heating gave the lowest FFA content (3.91%), whereas microwave-heating and infrared-radiation had similar FFA content with a mean value of 5.53%.

Lipase is the major cause of RB lipid deterioration i.e., hydrolytic oxidation. The changes in lipase activity as a result of stabilization treatments are also shown in Figure 1. Among the treatments, microwave-heating for 2 min at 650 Watt resulted in the most inefficient stabilization (78.6% relative enzymatic activity was remained). Dry-heating at 140 °C for 40 min showed better stabilization results (lowest content of liberated FA), with remaining lipase activity at ≈58.5%; moreover, infrared-radiation at the same temperature for 15 min gave even lower residual lipase activity of 34.8%. Yu et al. [47] compared 11 RB stabilization methods and found that among various heating treatments, autoclaving exerted the best inactivation effect, yielding ≈10.7% relative activity of lipase, whereas other heating methods resulted in about 30–35% residual lipase activities, except dry-heating (68.9%). However, although autoclaving was the most effective method to inactivate the lipase of RB, it is not easily applicable at an industrial scale. Furthermore, ultraviolet irradiation was found to be a promising alternative (convenient and energy-saving) stabilization method without influencing oil quality and nutrient content.

### 3.2. Nutritional Profile

The proximate analysis of untreated and heat-treated of RB samples, reflecting the major nutritional components, is presented in Table 1. As expected, the moisture content of all heat-treated RB samples is significantly lower (*p* < 0.05) than the untreated sample. Among the heat-treated samples, dry-heating significantly decreased the moisture content at the lowest level compared to microwave treatment, showing the highest level, whereas the infrared-radiation treatment gave an intermediate moisture content. The protein content was not affected significantly by the method of stabilization, ranging between 18.13 and 18.51%. However, stabilization by all heating methods increased significantly (*p* < 0.05) the fat and ash contents, when the data were expressed on a dry basis. The increased fat in heat-treated RB may be attributed to structure loosening of the treated samples, permitting a more efficient fat extractability by the organic solvent [21]. As for the carbohydrate content of the RB samples, there seems to be a very slight reduction (*p* < 0.05) in all heat-treated materials.

### 3.3. Fatty Acid Profile

The fatty acid composition of untreated and heat-stabilized RB oils (Table 2) has been determined to assess the effect of stabilization treatment. In general, RB oil is rich in polyunsaturated (41.70–42.60%), followed by monounsaturated (37.14–37.68%) and saturated fatty acids (19.50–20.03%). Palmitic (16.30–16.60%), oleic (36.30–36.80%), and linoleic acids (40.05–40.91%) were found to be the three dominant fatty acids, as previously reported in the literature [13,14,15]. The rations of polyunsaturated/saturated FAs (PUFA/SFA) and ω6/ω3 for untreated RB were 2.12 and 24.64, respectively, while the heat-treated RB samples were in the range of 2.08–2.19 and 24.35–26.66, respectively, without any significant change for the heated RB samples compared to the untreated RB. According to the nutritionally recommended values for the PUFA/SFA ratio (higher than 0.45) and ω6/ω3 ratio (a range between 4:1 and 10:1, due to differing opinions in the literature) [48], it can be concluded that the PUFA/SFA ratio of RB is beneficial to health, whereas the ω6/ω3 ratio does not comply with a desired good nutritional index [49].

The effect of heat treatments on the fatty acid composition of RB was found to be statistically insignificant (*p* > 0.05), as already observed by Yilmaz et al. [14] and Irakli et al. [15]. However, for infrared-radiation treatment, a slight increase in polyunsaturated and a decrease in monounsaturated and saturated fatty acids were noted compared to the untreated sample. Similarly, Ramezanzadeh et al. [13] found no significant differences for the fatty acids between stabilized RB by microwave-heating and the raw RB.

### 3.4. Color Parameters

**Figure 2:**
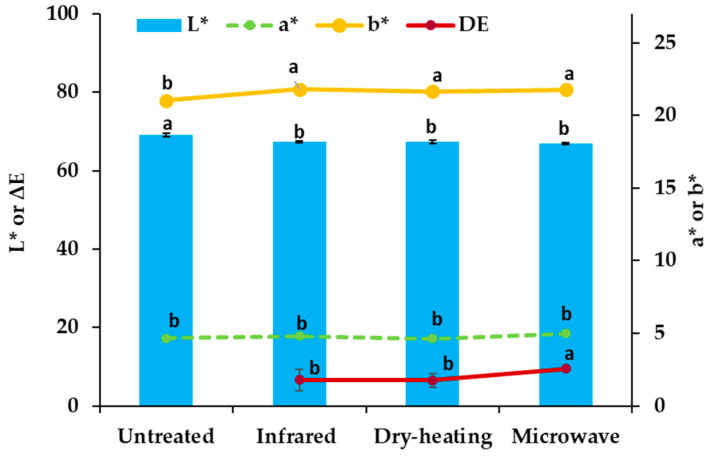
Effect of three different heating treatments (infrared-radiation, dry-heating, and microwave-heating) on color values of RB samples; values on bars or lines with specified by different letter are significantly different from each other (*p* < 0.05).

Color is an important quality determinant, influencing consumer preference for certain food products. The effects of different heating treatments on the color changes of RB are shown in Figure 2. The color values (L* and b*) were affected significantly (*p* < 0.05) by all three heating methods; however, no differences were noted among the heating treatments (*p* > 0.05). The RB stabilized with microwave-heating exhibited the brightest brown color, as evidenced from the highest ΔE* values among all stabilized RB samples. The color parameters of the stabilized RB samples were comparable to those of RB stabilized by other methods in previous studies; Rodchuajeen et al. [50] reported color values in the range of 62–64 for L*, 5.5–5.9 for a*, 18.2–19.1 for b*, and 6.1–8.2 for ΔE* for stabilized RB samples by different moving-bed drying methods.

### 3.5. Antinutritional Components

Although the increased consumption of cereal bran in the diet is encouraged by nutritionists to increase overall fiber intake, no consideration of the antinutritional components has ever been, although antinutrients are known to impair food digestion and absorption. The antinutritional composition of the RB samples before and after heat treatments is presented in Table 3. Stabilization treatments significantly reduced the antinutritional components. Overall, the highest phytate, oxalate, saponins, and trypsin inhibition levels were found in untreated RB, whereas heating treatments reduced all these antinutritional components. Phytate has been considered as an antinutrient because of its ability to interact with minerals, proteins, and starch, resulting in insoluble complexes that modify the functionality, digestion, and absorption of these food components [51]. The phytate content of untreated and treated RB samples ranged between 20.04 and 27.08 mg/g dw. Similar values are reported by Kaur et al. [52], although a large variation in phytate content is often found in the literature due to genotypic and environmental effects or different extraction rates adopted upon milling of rice grains. In the present study, the greatest reduction in phytate content was noted for the microwave stabilized RB (≈26.0%) followed by the infrared-radiation treated (≈22.2%) and the dry-heating treated RB (≈19.9%); however, no differences were observed among the heated-treated RB samples (*p* > 0.05). Previous work by Sharma et al. [21] showed a significant reduction in phytate content during the extrusion processing of RB. Similarly, Khan et al. [26] reported that several stabilization treatments significantly reduce the phytate content of RB. The phytic acid reduction by heating treatments may be partially due to the heat labile nature of phytic acid and the formation of insoluble complexes between phytate and other components [53].

Oxalate is also considered as an antinutritional component, as it can form non-absorbable salts with sodium, calcium, and ammonium ions, rendering these minerals unavailable [54]. High intakes of soluble oxalate may cause calcium oxalate crystallization and the formation of kidney stones (nephrolithiasis) in the urinary tract [55]. The effect of heating treatments on the oxalate content of RB is indicated in Table 3. Significant variation was noted in oxalate content among the RB samples. The oxalate content of untreated RB was 6.52 mg/g dw, which was reduced to 4.96, 4.25, and 5.80 mg/g dw with the infrared-radiation, microwave-heating, and dry-heating, respectively. Among the different heating treatments, microwave-heating was found most effective in reducing the oxalate content in RB by 34.9%. Kaur et al. [52] also reported a lower level of oxalate content subjected to extrusion cooking, compared to its untreated counterpart.

Trypsin inhibitors, being low molecular weight proteins, were significantly inactivated (*p* < 0.05) by all heating treatments employed, as illustrated in Table 3. The trypsin inhibition was minimum in the dry-heated RB, followed by microwave-heated and infrared-radiation-treated RB samples; i.e., dry-heating, microwave-heating, and infrared-radiation was effective in reducing the trypsin inhibitor activity by ≈32.5, 31.0, and 27.4%, respectively. It has been reported that heat treatments such as microwave treatment, cooking, and autoclaving inactivate the trypsin inhibitors as a result of denaturation of these heat-labile proteins [56]. It has been suggested that reactions involving deamidation (splitting of covalent bonds) and the destruction of disulfide bonds might be responsible for the thermal inactivation process [52].

Saponins have the ability to bind proteins, enhancing protein stability against heat denaturation and decreasing the susceptibility of proteins to proteases. They may also cause gastrointestinal lesions, entering into the blood stream and hemolyzing the red blood cells [57]. The data displayed in Table 3 show the effect of stabilization on the saponin content of RB. All heating treatments resulted in a significant reduction of the saponin content, with the dry and microwave-heating presenting the maximum percentage reduction in saponin content at ≈30.5 and 26.6%, respectively, whereas the infrared radiation was less effective (reduction ~12.0%). The loss of saponins during microwave heating might be attributed to the thermo-labile nature of these compounds.

Although tannins form tannin–protein complexes leading to the inactivation of digestive enzymes and protein insolubility, they are considered effective in lowering blood glucose levels by delaying intestinal glucose absorption, thus delaying the onset of insulin-dependent diabetes mellitus [58]. In the present study, dry-heating, microwave processing, and infrared-radiation did not seem to reduce the total tannin content, in contrast to all other antinutritional components studied. Similarly, Sahni and Sharma et al. [59] also found that thermal processing treatments on alfalfa seeds showed less reduction in tannin content than other treatments. Moreover, Osman [60] found that the tannins content increased in roasted or cooked lablab beans due to the inhibition of polyphenol oxidase after heat treatment. However, Deng et al. [61] reported a significant reduction of tannins in buckwheat grains after cooking and microwave treatment.

### 3.6. Bioactive Components

The principal bioactive compounds that contribute to the promising health-related benefits of RB comprise of phenolic compounds, tocols, and sterol derivatives (particularly γ-oryzanol). The phenolic compounds in RB exhibit antioxidant activity and may reduce free radical-mediated cellular damage. The TPC of each of the stabilized RB samples was determined using the Folin–Ciocalteu method in both free and bound forms. Our results demonstrate that total TPC decreased (*p* < 0.05) as a result of the thermal stabilization of RB (Table 4), with significant differences (*p* < 0.05) among heat treatment applied. Untreated RB had the highest total TPC, followed by microwave-heated, infrared-radiated, and dry-heated RB samples. The free and bound TPC decreased by 16.5% and 10.3% after dry-heating, by 12.3% and 11.1% after infrared-radiation, and by 11.4% and 8.1% after microwave-heating treatment, respectively. Similar observations have been made by Rodchuajeen et al. [50], showing that heat processing can reduce the polyphenolic components of RB. In contrast, Saji et al. [62] found that stabilized RB by dry-heating had higher TPC than the untreated material. This discrepancy may be due to the fact that different factors, such as extraction parameters, varietal differences, bran fraction, and environmental conditions during the growing season may have a compositional impact and modulate the effects of the stabilization treatments.

The major lipophilic fractions of RB are tocopherols, tocotrienols (known as tocols), and γ-oryzanol that are characterized as the strongest antioxidants in RB [9]. According to the results presented in Table 4, the contents of tocotrienols and tocopherols in heat-treated RB were significantly lower than the untreated material (*p* < 0.05), which was presumably due to the degradation of the heat-sensitive antioxidants, with a loss of 15.8 and 20.5% for tocopherols and tocotrienols, respectively. On the contrary, γ-oryzanol was not reduced significantly (*p* < 0.05) by all heat treatments, as it has been reported to be a relatively thermostable antioxidant [63]. Untreated RB had the highest γ-oryzanol content, whereas the microwave-heated RB the lowest concentration among all the heat-treated RB samples. Similar results have been reported in the study of Lakkakula et al. [64] in which ohmic heat processing was adopted for RB stabilization.

The effects of heat treatments on the antioxidant activity of RB were evaluated by three different methods, and the results are given in Table 4. Generally, greater antioxidant activity was observed in the free phenolic extract, as evaluated by the three tests (DPPH, ABTS, and FRAP). The DPPH free radical method is being used extensively to evaluate reducing substances. It has been noted that the DPPH radical scavenging activity of free RB phenolics was on average 2.6 times higher than those of the extracts of bound phenolics. A significant decrease in DPPH radical scavenging activity of the free phenolic fractions as well as total phenolics appeared upon heat treatment; nevertheless, the DPPH values of the bound fractions were not altered significantly, except in the case of the infrared-radiation and the dry-heating treatments. Such reductions go in parallel with the reduction in TPC and could be attributed to the formation of an irreversible covalent bond between liberated phenolic compounds and proteins [65].

A similar trend was observed for ABTS radical scavenging activity in the case of extracts of free phenolics, as the ABTS scavenging capacity decreased for all heat-stabilized RB samples (Table 4). However, for the bound phenolic extracts, the ABTS scavenging capacity increased after heat-stabilization, particularly for samples subjected to dry and microwave heat treatments (≈2.4 folds). This could be due to the differences in the type of radicals and phenolic compounds in both extracts. The presence of other phenolic compounds and non-phenolic antioxidants in the free extracts could also contribute to the antioxidant potential of these materials. Despite the large differences observed between free and bound phenolic extracts, which is in contrast to the DPPH assay, higher antioxidant activity was also noted in a study by Saji et al. [62] in which microwave and dry-heating methods were adopted for the RB stabilization. Overall, among all stabilization treatments, dry-heating and microwave-heating led to 23.9 and 34.2% increase in total ABTS value, respectively, while the infrared-radiation did not bring a significant change.

For the bound phenolic extracts, the antioxidant capacity of all stabilized RB samples, using the FRAP test, was increased compared to the untreated RB, and this was more pronounced to microwave-treated RB sample (Table 4). Some differences in the antioxidant activity values were also noted among the free phenolic extracts; the sample treated with infrared radiation exhibited the highest value for antioxidant capacity. The enhanced antioxidant activity of heat-treated RB samples may be due to the generation and accumulation of Maillard-derived melanoidins at high temperatures [66].

### 3.7. Functional Properties

**Figure 3:**
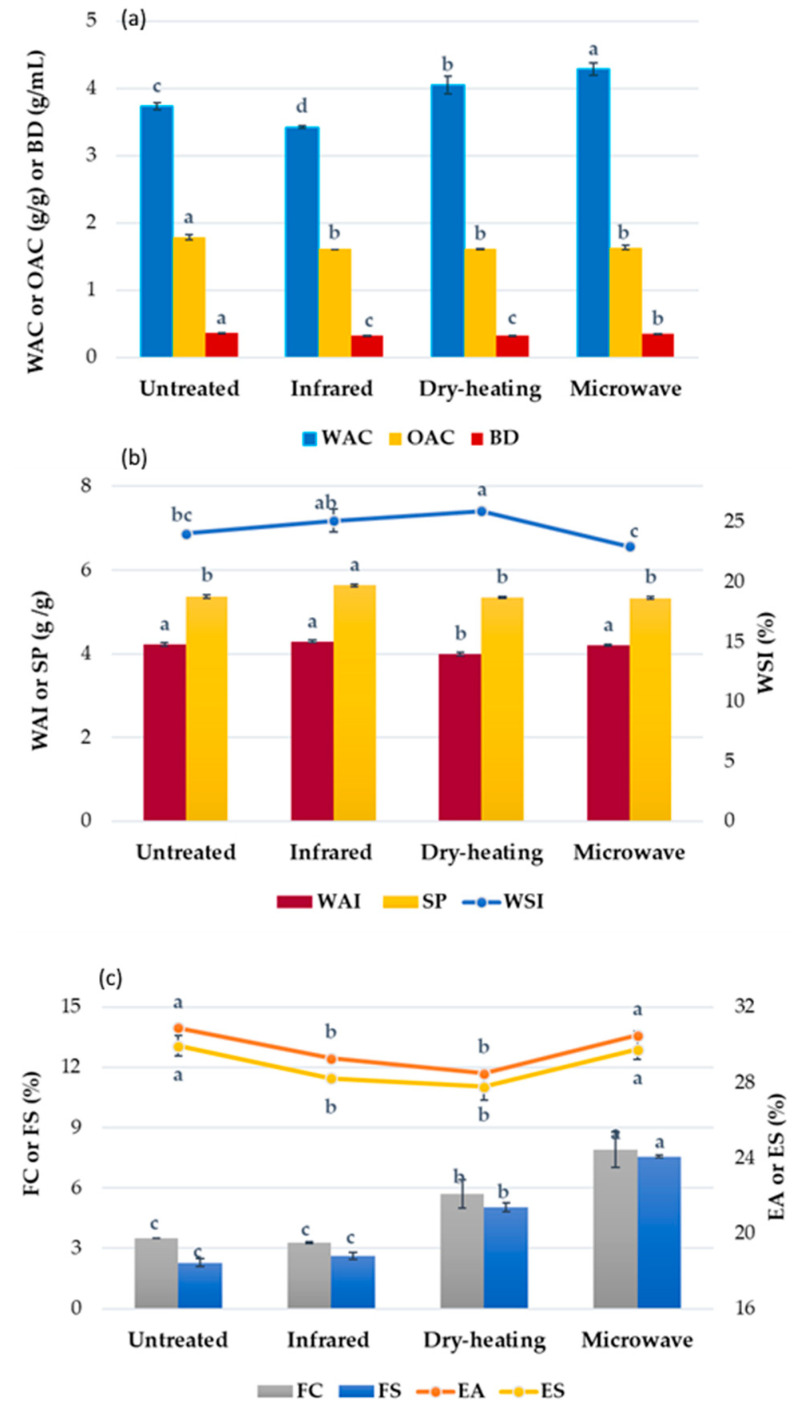
Effect of three different heating treatments (infrared-radiation, dry-heating, and microwave-heating) on WAC, OAC and BD (**a**), WAI, WSI and SP (**b**) and FC, FS, EA and ES (**c**) of RB samples; values followed by the same letter for the same physicochemical parameter are not significantly different (*p* > 0.05). WAC: Water Absorption Capacity, OAC: Oil Absorption Capacity, BD: Bulk Density, WAI: Water Absorption Index, SP: Swelling Power, WSI: Water Solubility Index, FC: Foaming Capacity, FS: Foaming Stability, EA: Emulsifying Activity, ES: Emulsion Stability.

The bulk density (BD) is a key factor for the storage, transportation, and processing of dry powders in food product formulations. In our study, the BD of RB samples varied from 0.33 to 0.36 g/mL. The stabilization by all heat treatments slightly decreased the BD of RB. The lower values for BD (*p* < 0.05) were observed for the infrared-treated and dry-heated RB (Figure 3a), which was presumably due to some structural collapse of the material, leading to decreased porosity. A slightly higher BD in the microwave-heated RB reflects a more compact structure for these materials.

The functional properties of RB are important for its technological interest and physiological effects. The hydration properties of the RB dietary fiber determine their optimal usage levels in composite food matrices, since a desirable texture should be always retained [67]. The water absorption capacity (WAC) of RB samples ranged from 3.42 to 4.29 g/g dw (Figure 3a), which is within the range of 1.49–4.72 g/g, which is considered critical in viscous food dispersions [68]. The results indicate that the WAC of RB was affected significantly (*p* < 0.05) by the application of different methods of stabilization. Specifically, the WAC of stabilized RB by dry-heating and microwave-heating improved by 8.4 and 14.8% (*p* < 0.05), respectively, making it a more suitable ingredient in foods where a greater water absorption is targeted (e.g., baked goods). However, the infrared-radiation treatment decreased significantly the WAC of RB compared to the untreated sample. In general, the relatively high WAC of the RB materials might be due to the presence of the hydroxyl group bearing polysaccharide components and the polar amino acids at the bran particle–water interface [27]. Similarly, Rafe et al. [68] found that the stabilization of RB by extrusion improved up to 20% the WAC; however, Khan et al. [27] indicated that the WAC of RB stabilized by dry-heating and microwave-heating was not significant (*p* > 0.05) as a result of the applied thermal treatment.

Oil absorption capacity (OAC) is another important functional property, which is related to mouthfeel perception of the final product. A high OAC is essential in food systems such as processed meats (sausages), cake batters, mayonnaise, and salad dressings. According to the results of Figure 3a, the heat-treated RB samples showed slightly less strength for binding oil as compared to their counterpart. This might imply decreased surface hydrophobicity following the heat treatment. A lower hydrophobicity of the stabilized RB particles would not enhance the interactions between the fiber matrix of the RB and the oil, resulting in decreased oil absorption capacity [69].

The water absorption index (WAI) is the amount of water absorbed by starch or other particulate materials after swelling in an excess of water. In the case of starch, it can be related to starch gelatinization, which is greatly affected by the temperature and moisture content of the heated raw material [70]. The WAI was not affected significantly (*p* > 0.05) with the infrared-radiation and microwave-heating of RB stabilization (Figure 3b). Among all heat treatments applied, the dry-heating brought about the highest influence on WAI, leading to a 5.4% decrease in WAI of stabilized RB. The water solubility index (WSI) measures the amount of solubles (mostly low molecular weight material and water-soluble polysaccharides) released from a starch-containing material after thermal treatment. It is generally used as an indicator of material phase change (e.g., starch gelatinization) and/or some degradation of molecular components present in the heated particles [69]. The increase of WSI with heat treatment was more pronounced in the dry-heated RB, which was followed by the infrared-radiated RB, whereas the microwave-heated RB showed similar WSI with the untreated sample, as shown in Figure 3b. This may be due to the higher moisture content of RB treated by microwaves compared to the other samples [70]. Similar results were also reported for RB stabilized by extrusion cooking [71] and by microwave-heating [72]. A similar trend to WAI was also observed for swelling power (SP), indicating rather insignificant effects (*p* > 0.05) for the SP in all thermally stabilized RB, compared with the untreated RB; however, infrared radiation treatment increased significantly (*p* < 0.05) the SP of RB.

The foaming capacity (FC) and foaming stability (FS) of untreated and heat-stabilized RB samples are presented in Figure 3c. Generally, the RB has a low FC that may be associated with the presence of amphiphilic lipids in the RB, since these are more easily adsorbed at the interface than proteins, influencing the strength and elasticity of the film and, therefore, its ability to incorporate air [73]. It is noticed that the heat stabilization of RB improved the FC compared to the untreated RB, particularly in the case of dry-heating and microwave treated samples; i.e., FC values were improved from 3.51% (untreated) to 5.70 and 7.92% after treatment by dry-heating and microwave-heating, respectively. Proteins also play a significant role in forming a stable air bubble because of their amphiphilic nature; i.e., the protein structure gets rapidly unfolded to form a cohesive macromolecular layer at the air–water interface [72]. Heat treatment could partially unfold the protein chains, making them easier to absorb at the air–water interface, and as a result, it leads to an increase in FC. Similarly, Rafe et al. [68] reported that extruded RB had a higher FC than unstabilized RB, and Zhu et al. [74] also found that the RB protein following high-pressure treatment enhanced its FC. Instead, Khan et al. [27] found that extended heat application impairs the foaming properties of protein products, whereas partial enzymatic hydrolysis improves the interfacial properties of these biomolecules. The decrease in the FC of the RB protein by infrared treatment might be due to its thermal denaturation, making the diffusion and adsorption of the protein components at the air–water interface more difficult [75]. FS refers to the ability to maintain the air bubble against breaking or collapsing. Similar to FC, FS increased with heat treatment following dry-heating and microwave-heating of the RB with an increase of 121 and 232%, respectively, as compared with the untreated RB. However, there were no significant differences (*p* > 0.05) in the FS between untreated and stabilized RB by infrared-radiation. An improvement in FS might be related to enhanced protein–protein interactions (aggregation) upon heat treatment, bringing about a thick proteinaceous film around the air bubbles [76].

The emulsifying activity (EA) is mainly dependent on the diffusion of surface active constituents at the oil–water interfaces. Τhe effect of heat stabilization of RB on EA and emulsion stability (ES) of RB is shown in Figure 3c. Dry-heating and infrared-radiation treatments of RB decreased the EA of RB (*p* < 0.05). Instead, microwave-heating treatment did not lead to significant changes in the EA of RB. The emulsions of all RB samples were very stable in the present study; the ES showed a similar trend among all samples to that of EA. In general, heating treatments decreased the surface activity and emulsion properties, which was probably due to protein denaturation in high temperatures [77]. Similar observations have been also reported by Capellini et al. [73] for rice bran defatted with alcoholic solvents.

## 4. Conclusions

The present study has demonstrated that infrared-radiation heating among other heat treatments such as dry-heating and microwave-heating could be an efficient method for inactivating lipase activity and prolonging the shelf-life of RB. It took a shorter processing time to effectively stabilize this dietary fiber source without affecting the nutritional profile, γ-oryzanol content, and ABTS radical scavenging activity while reducing the antinutrients and improving the ferric reducing antioxidant power. It also enhanced some functional properties (WSI and SP) of the heat-treated RB that may improve its use as a functional ingredient in food and cosmetics formulations. Instead, microwave stabilization was an effective treatment for improving the foaming and emulsifying capacities as well as water absorption of the RB. In addition, microwave treatment resulted in the largest reductions of antinutrients present in RB among all heat stabilization methods tested. Overall, among all studied methods, infrared-radiation heating appears a promising processing alternative to conventional dry-heating to stabilize RB, provided that appropriate optimization studies will be undertaken to examine the impact of the various operation parameters and conditions on the physicochemical, bioactivity, and functional properties of this material at a commercial scale level.

## Figures and Tables

**Table 1:** Proximate composition (% db) of rice bran (RB) samples before and after heat treatments.

Samples	Moisture	Protein	Fat	Ash	Carbohydrates
Untreated	10.93 ± 0.07 ^a^	18.39 ± 0.03 ^ab^	17.81 ± 0.31 ^b^	8.83 ± 0.03 ^b^	55.01 ± 0.34 ^a^
Infrared	5.48 ± 0.05 ^c^	18.13 ± 0.10 ^b^	18.76 ± 0.37 ^a^	8.94 ± 0.04 ^a^	54.42 ± 0.27 ^b^
Dry-heating	2.76 ± 0.00 ^d^	18.51 ± 0.10 ^a^	18.53 ± 0.04 ^a^	9.00 ± 0.02 ^a^	53.97 ± 0.12 ^b^
Microwave	8.30 ± 0.01 ^b^	18.37 ± 0.10 ^ab^	18.74 ± 0.18 ^a^	8.96 ± 0.01 ^a^	53.94 ± 0.02 ^b^

Values are means ± standard deviation (*n* = 3), while different letters for values in each column indicate significant differences (*p* < 0.05); db: dry basis.

**Table 2:** Fatty acid profile of RB samples before and after heat treatments (% of the total fatty acids).

Fatty Acids	Untreated	Infrared	Dry-Heating	Microwave
Myristic acid (C14:0)	0.36 ± 0.01 ^c^	0.41 ± 0.01 ^a^	0.38 ± 0.01 ^b^	0.35 ± 0.01 ^bc^
Palmitic acid (C16:0)	16.30 ± 0.30 ^a^	16.30 ± 0.50 ^a^	16.55 ± 0.15 ^a^	16.60 ± 0.10 ^a^
Stearic acid (C18:0)	1.30 ± 0.01 ^a^	1.19 ± 0.03 ^b^	1.31 ± 0.01 ^a^	1.30 ± 0.01 ^a^
Oleic acid (C18:1, *cis*-9)	36.75 ± 0.15 ^a^	36.30 ± 0.70 ^a^	36.70 ± 0.10 ^a^	36.80 ± 0.10 ^a^
Linoleic acid (C18:2 *cis*-9,12), n6	40.29 ± 0.22 ^a^	40.91 ± 1.06 ^a^	40.05 ± 0.25 ^a^	40.25 ± 0.15 ^a^
α-Linolenic acid (C18:3, *cis*-9,12,15), n3	1.64 ± 0.02 ^a^	1.54 ± 0.12 ^a^	1.64 ± 0.01 ^a^	1.65 ± 0.02 ^a^
Arachidic acid (C20:0)	0.64 ± 0.01 ^a^	0.51 ± 0.01 ^c^	0.58 ± 0.01 ^b^	0.57 ± 0.01 ^b^
*cis*-11-Eicosenoic acid (C20:1)	0.55 ± 0.01 ^ab^	0.52 ± 0.01 ^c^	0.55 ± 0.01 ^a^	0.53 ± 0.01 ^bc^
Behenic acid (C22:0)	0.27 ± 0.01 ^b^	0.28 ± 0.01 ^b^	0.28 ± 0.01 ^a^	0.29 ± 0.01 ^a^
Lignoceric acid (C24:0)	0.76 ± 0.01 ^b^	0.71 ± 0.02 ^c^	0.82 ± 0.02 ^a^	0.75 ± 0.01 ^b^
Saturated fatty acids (SFA)	19.74 ± 0.28 ^a^	19.50 ± 0.55 ^a^	20.03 ± 0.18 ^a^	20.02 ± 0.09 ^a^
Monounsaturated fatty acids	37.64 ± 0.14 ^a^	37.14 ± 0.70 ^a^	37.63 ± 0.08 ^a^	37.68 ± 0.10 ^a^
Polyunsaturated fatty acids (PUFA)	41.93 ± 0.20 ^a^	42.60 ± 0.98 ^a^	41.70 ± 0.25 ^a^	41.92 ± 0.17 ^a^
PUFA/SFA	2.12 ± 0.04 ^a^	2.19 ± 0.11 ^a^	2.08 ± 0.03 ^a^	2.09 ± 0.02 ^a^
ω6	40.36 ± 0.16 ^a^	40.92 ± 1.04 ^a^	40.21 ± 0.17 ^a^	40.30 ± 0.13 ^a^
ω3	1.64 ± 0.02 ^a^	1.54 ± 0.12 ^a^	1.64 ± 0.01 ^a^	1.65 ± 0.02 ^a^
ω6/ω3	24.64 ± 0.40 ^a^	26.66 ± 2.66 ^a^	24.59 ± 0.07 ^a^	24.35 ± 0.15 ^a^

Minor fatty acids were lauric (C12:0), pentadecanoic (C15:0), heptadecanoic (C17:0), *cis*-10 heptadecenoic (C17:1), linolelaidic (C18:2, *trans*-9,12), erucic (C22:1, *cis*-13), tricosanoic (C23:0). Means with same superscript letter within the same line are not significantly different (*p* > 0.05).

**Table 3:** Antinutritional composition of RB samples before and after heat treatments.

Samples	Phytate (mg/g dw)	Oxalate (mg/g dw)	Saponins (mg/g dw)	Tannins (mg/g dw)	Trypsin Inhibition (TUI/g dw)
Untreated	27.08 ± 0.73 ^a^	6.52 ± 0.15 ^a^	89.70 ± 0.28 ^a^	3.39 ± 0.16 ^a^	14.93 ± 0.79 ^a^
Infrared	21.06 ± 0.46 ^b^	4.96 ± 0.07 ^c^	78.98 ± 1.59 ^b^	3.27 ± 0.02 ^a^	10.84 ± 0.37 ^b^
Dry-heating	21.70 ± 0.11 ^b^	5.80 ± 0.04 ^b^	62.35 ± 2.57 ^c^	3.37 ± 0.06 ^a^	10.08 ± 0.10 ^b^
Microwave	20.04 ± 1.00 ^b^	4.25 ± 0.07 ^d^	65.87 ± 0.82 ^c^	3.44 ± 0.14 ^a^	10.31 ± 0.38 ^b^

Values are mean ± standard deviation, while same letter along a column indicates no significant differences at *p* > 0.05; dw, dry-weighted.

**Table 4:** Bioactive components of RB samples before and after heat treatments.

Samples	Fractions	Untreated	Infrared	Dry-Heating	Microwave
TPC (mg GAE/g dw)	Free	5.99 ± 0.05 ^a^	5.15 ± 0.06 ^c^	5.00 ± 0.01 ^d^	5.31 ± 0.01 ^b^
	Bound	1.80 ± 0.02 ^a^	1.60 ± 0.06 ^b^	1.62 ± 0.03 ^b^	1.65 ± 0.04 ^b^
	Total	7.79 ± 0.03 ^a^	6.75 ± 0.01 ^c^	6.61 ± 0.02 ^d^	6.96 ± 0.02 ^b^
Tocols (mg 100/g dw)	T3s	13.45 ± 0.20 ^a^	11.42 ± 0.31 ^b^	10.96 ± 0.23 ^b^	11.59 ± 0.41 ^b^
	Ts	3.22 ± 0.03 ^a^	2.54 ± 0.01 ^b^	2.58 ± 0.01 ^b^	2.57 ± 0.26 ^b^
	Total	16.67 ± 0.16 ^a^	13.96 ± 0.31 ^b^	13.54 ± 0.24 ^b^	14.16 ± 0.67 ^b^
γ-Oryzanol (mg/g dw)		2.44 ± 0.04 ^a^	2.37 ± 0.03 ^ab^	2.40 ± 0.01 ^ab^	2.32 ± 0.13 ^ab^
DPPH (mg TE/g dw)	Free	7.75 ± 0.06 ^a^	6.79 ± 0.14 ^b^	6.69 ± 0.05 ^b^	6.85 ± 0.09 ^b^
	Bound	2.72 ± 0.19 ^a^	2.17 ± 0.02 ^b^	2.39 ± 0.15 ^b^	2.79 ± 0.08 ^a^
	Total	10.46 ± 0.25 ^a^	8.96 ± 0.12 ^c^	9.08 ± 0.20 ^c^	9.64 ± 0.01 ^b^
ABTS (mg TE/g dw)	Free	14.73 ± 0.11 ^a^	12.92 ± 0.18 ^c^	12.69 ± 0.30 ^c^	13.61 ± 0.11 ^b^
	Bound	5.35 ± 0.33 ^c^	5.05 ± 0.49 ^c^	12.65 ± 0.41 ^b^	13.03 ± 0.29 ^a^
	Total	20.73 ± 0.48 ^c^	18.27 ± 0.34 ^d^	25.70 ± 0.72 ^b^	27.82 ± 0.43 ^a^
FRAP (mg TE/g dw)	Free	6.61 ± 0.04 ^b^	7.13 ± 0.17 ^a^	6.58 ± 0.06 ^b^	5.01 ± 0.01 ^c^
	Bound	2.46 ± 0.08 ^c^	2.55 ± 0.03 ^c^	3.69 ± 0.03 ^b^	3.98 ± 0.16 ^a^
	Total	9.08 ± 0.12 ^c^	9.68 ± 0.20 ^b^	10.27 ± 0.10 ^a^	8.99 ± 0.14 ^c^

Values are mean ± standard deviation, while same letter along a line indicates no significant differences (*p* > 0.05). TPC: total phenolic content.
